# Toward *In Vivo* Transdermal pH Sensing with a Validated Microneedle Membrane Electrode

**DOI:** 10.1021/acssensors.0c02397

**Published:** 2021-02-10

**Authors:** Juan José García-Guzmán, Clara Pérez-Ràfols, María Cuartero, Gastón A. Crespo

**Affiliations:** Department of Chemistry, School of Engineering Science in Chemistry, Biochemistry and Health, Royal Institute of Technology, KTH, Teknikringen 30, SE-100 44 Stockholm, Sweden

**Keywords:** ion-selective electrodes, pH sensing, in vivo monitoring, microneedle transdermal sensing, validation

## Abstract

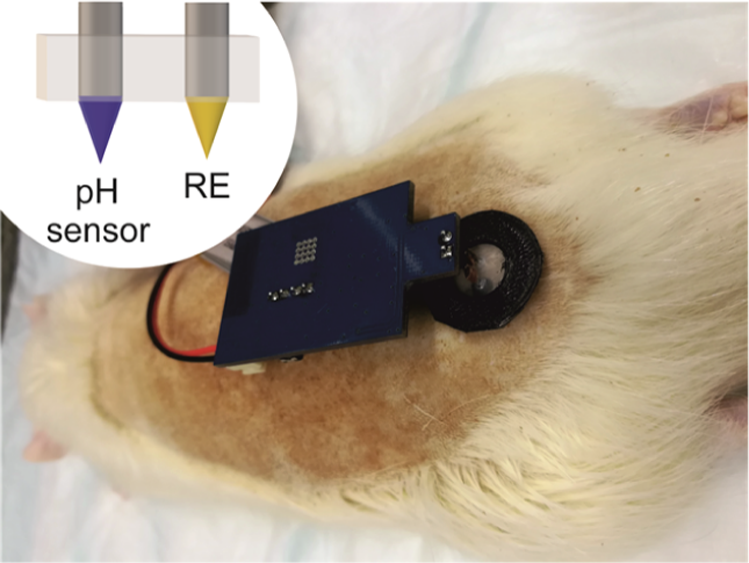

We present herein the most complete characterization of microneedle (MN) potentiometric sensors for pH transdermal measurements for the time being. Initial *in vitro* assessment demonstrated suitable analytical performances (e.g., Nernstian slope, linear range of response from 8.5 to 5.0, and fast response time) in both buffer media and artificial interstitial fluid (ISF). Excellent repeatability and reproducibility together with adequate selectivity and resiliency facilitate the appropriateness of the new pH MN sensor for transdermal ISF analysis in healthcare. The ability to resist skin insertions was evaluated in several *ex vivo* setups using three different animal skins (i.e., chicken, pork, and rat). The developed pH MN sensor was able to withstand from 5 to 10 repetitive insertions in all the skins considered with a minimal change in the calibration graph (<3% variation in both slope and intercept after the insertions). *Ex vivo* pH measurements were validated by determining the pH with the MN sensor and a commercial pH electrode in chicken skin portions previously conditioned at several pH values, obtaining excellent results with an accuracy of <1% and a precision of <2% in all cases. Finally, pH MN sensors were applied for the very first time to transdermal measurements in rats together with two innovative validation procedures: (i) measuring subcutaneous pH directly with a commercial pH microelectrode and (ii) collecting ISF using hollow MNs and then the pH measurement of the sample with the pH microelectrode. The pH values obtained with pH MN sensors were statistically more similar to subcutaneous measurements, as inferred by a paired sample *t*-test at 95% of confidence level. Conveniently, the validation approaches could be translated to other analytes that are transdermally measured with MN sensors.

Microneedle (MN) sensors are attracting increasing attention linked to the need for new wearable diagnostic tools within the healthcare system.^1,2^ Indeed, the intrinsic ability of MN sensors to perform minimally invasive on-body and real-time measurements is crucial to accelerate the provision of meaningful observations in view of the next generation of personalized and preventive medicine. Unlike other formats of wearable chemical sensors that target “excretable” biological fluids such as sweat, tears, and saliva (e.g., sweat bands and patches, smart watches, glasses, and contact lenses),^3−6^ MN sensors are primarily conceived for transdermal monitoring of interstitial fluid (ISF). Advantageously, the ISF composition has claimed to be very similar to blood, as a consequence of the existing equilibrium between ISF and plasma carried out by several small molecules (e.g., albumin, CO_2_, and phosphates).^2^ This similarity in composition is appealing for clinical purposes in order to substitute current (and inconvenient for the patient) blood analysis while providing the same physiological outcomes. In addition, biofouling in ISF is known to be lower than in blood due to lower concentration of proteins and other large molecules.^2,7,8^

The analysis of ISF entails several challenges rarely addressed in the literature such as resistance to skin penetration and strict analytical validation (mostly absent) of the obtained analyte levels.^2^ It is essential to unequivocally demonstrate that the chemical sensing element embedded in the MN is not altered during skin insertion prior to claim any suitability for further *in vivo* applications; otherwise, the developed device may create unreal expectations. Special attention should be paid when the MN is externally modified with the sensing element because this is totally exposed to the skin penetration process. Therefore, external modification presents an increasing probability of detachment/alteration together with incremental cytotoxicity risk in the monitored subject compared to internal modification of the MN.^2^

Concerning the analytical validation of transdermal measurements through MNs, the main problem lies in the difficulty of extracting enough volume of ISF to be measured using a reference analytical technique. Several strategies have been reported for this purpose including hollow MNs, effusion, dialysis, sonication, and reverse iontophoresis.^2,9^ The latter has been demonstrated for the successful analysis of glucose but is not valid for all analytes because the iontophoresis process per se modifies the composition of the collected ISF.^9^ By contrast, extraction with hollow MNs preserves ISF composition as far as pertinent precautions are fulfilled to avoid sample evaporation and/or external contamination, which is a common issue whatever the collection method. The main limitation of ISF collection with MNs is that the volume is very low, usually less than 10 μL.^10,11^ Thus, in view of this problem, most *in vivo* studies using MN sensors attempt a validation by inducing changes in analyte concentration^12,13^ or comparing with concentration in blood, which has been widely applied in the case of MNs for glucose detection.^14,15^ Evidently, these strategies cannot be considered as pure analytical validation methods because they are either qualitative or do not consider the same fluid.

A search into the literature reveals that *in vivo* implementation of MN chemical sensors primarily targets glucose, with pH being the second analyte most widely detected.^2^ While the interest of measuring glucose to manage diabetes is undeniable, it has been proved that acid/base disorders are closely related to many diseases such as renal failure, ischemia, multiple sclerosis, and psychiatric disorders. Therefore, monitoring *in vivo* dynamic changes in pH has been claimed to be highly relevant from a clinical perspective.^16^ While a great number of pH MN sensors can be found in the literature, with electrochemical readout but also others,^2,17,18^ to the best of our knowledge, only few works reported on real applications involving the *in vivo* determination of pH and transdermal measurements in ISF totally lack.

In this context, Mani et al. analyzed pH in mouse cerebrospinal fluid (CSF) and bladders using tungsten-modified MNs (W/ZnO)^16^ while Zuliani et al. developed an iridium oxide (IrOx) MN sensor for mapping pH distributions in rat heart.^19^ Zhou et al. developed a pH sensor based on molybdenum disulfide (MoS_2_) nanosheets and polyaniline (PANI)-functionalized acupuncture needles for real-time monitoring of pH changes in rat brain.^20^ However, these papers did not cover the determination of pH in ISF and lack the evaluation of a possible detachment of the sensing element with the transdermal use of the MN and how this may affect the monitored individual. In this sense, it is important to mention that all the tungsten compounds are regarded as highly toxic compounds,^21^ whereas very little information is available for IrOx and the PANI-MoS_2_ tandem despite careful manipulation of these compounds being generally advised.^22,23^ In addition, the high-cost and high-temperature processing associated to metal oxide-based pH sensors is known to limit the fabrication of disposable MN sensors.^24^

In analogy to solid-contact electrochemical sensors already well-entrenched for pH detection in diverse samples, potentiometric ion-selective electrodes (ISEs) based on plasticized polymeric membranes should be good candidates toward effective transdermal monitoring of pH in ISF, beyond the use of inorganic coverings that are pH-responsive (i.e., W/ZnO, IrOx, and PANI-MoS_2_).^24,25^ However, to the best of our knowledge, this approach has not been translated to the MN configuration yet. Importantly, our group has recently demonstrated the huge potential of the ISE technology toward potassium detection in ISF.^26^ Commercially available stainless-steel solid MNs were externally modified, first with the solid ion-to-electron transducer and then with the ion-selective membrane (ISM), following a procedure adapted from the traditional all-solid-state ISE fabrication.^27^ The proposed MN technology demonstrated excellent resiliency to skin penetration and the absence of biofouling (in the time scale of hours).^26^ In addition, cytotoxicity studies using human dermal fibroblasts revealed a connection with the leaching of the ionophore from the ISM to the cell culture with any found toxic effect.^28^ Thus, while a toxic impact was detected in the 24–36 h experimental time frame for potassium and ammonium ISMs (based on valinomycin and nonactin, respectively), no effect appeared for other ionophores, including hydrogen ionophore I (typically used in pH sensors).^28^ In principle, these results pointed out the suitability of pH-responsive ISMs to be implemented into MN technology for transdermal ISF analysis. This approach is expected to exceed the sensor biocompatibility compared to already existing pH MN sensors commented above.^2,16,19,20^

We present herein the very first MN sensor applied to the transdermal detection of pH in ISF in rats. It herein shows a deep characterization and pioneering validation protocol for pH MN sensors, which is schematized in Figure 1 and could pave the way as a general strategy for any MN chemical sensor validation. In addition, the present work innovates toward proper inquiry of MN sensing technology through the *in vitro*, *ex vivo*, and *in vivo* journey aiming at reaching on-body measurements. Initial *in vitro* assessment is performed to evaluate the analytical performances of the developed pH MN sensors in controlled buffer media and artificial ISF (AISF). Subsequently, the ability of the MN sensor to perform transdermal measurements is evaluated using several animal skins and following two protocols. The first one compares pre- and postinsertion calibrations in the skin to ensure that the sensing element is not damaged. The second protocol aims at testing the accuracy of transdermal pH measurements in skin pieces conditioned at different pHs, that is, with known analyte concentration. Finally, pH MN sensors are tested in rats together with two different validation approaches based on subcutaneous measurements with a commercially available microelectrode and ISF extraction with bare hollow MNs. Advantageously, the procedure to fabricate and validate the pH MN sensors can be tailored for other ions and molecules, therefore prospecting fast advances in accurate multianalyte monitoring in ISF toward *in vivo* measurements in animals and humans.

**Figure 1:**
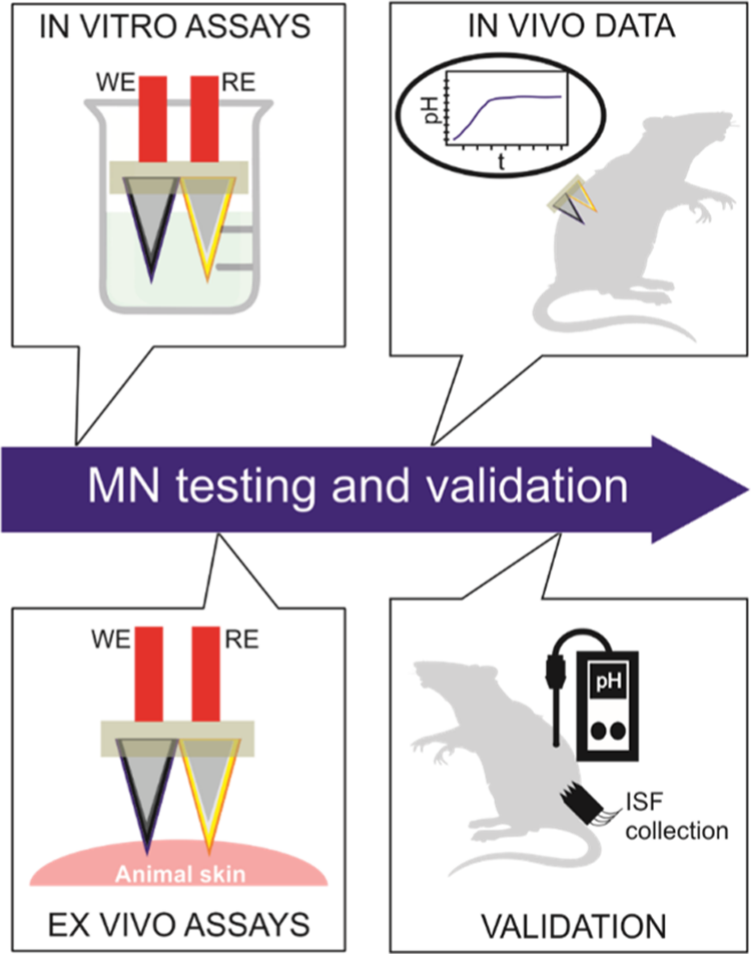
Scheme for the protocol used in the present paper for the characterization and validation of the pH MN patch.

## Experimental Section

### Preparation of the MN Patch for the Potentiometric Determination of pH

**Figure 2:**
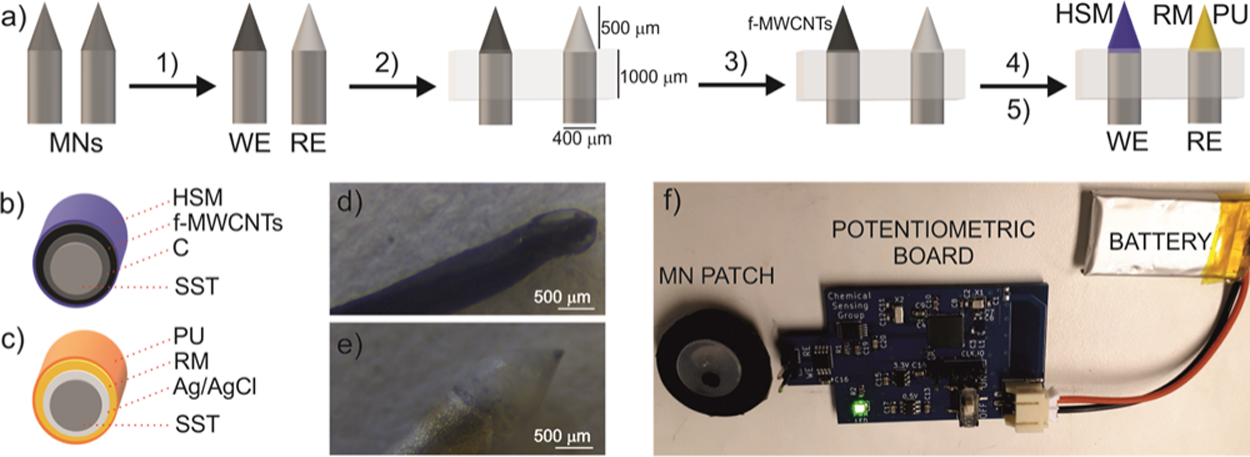
(a) Manufacturing procedure for the MN patch containing both the pH-selective electrode and reference electrode: (1) stainless-steel solid MNs are modified with carbon and Ag/AgCl ink coatings to further produce the working (WE) and reference electrode (RE), respectively; (2) modified MNs are assembled into the silicon substrate; (3) deposition of f-MWCNTs on top of the carbon ink in the WE, (4) deposition of the pH-selective membrane (HSM) in the WE; and (5) deposition of the RM and PU external layer in the RE electrode. (b) Cross-sectional image of the layers’ arrangement in the WE, namely, stainless-steel core, carbon ink, f-MWCNTs, and HSM. (c) Cross-sectional image of the layers’ arrangement in the RE: stainless-steel core, Ag/AgCl ink, RM, and PU. (d) Image obtained by optical microscopy for the WE. (e) Image obtained by optical microscopy for the RE. (f) Arrangement of the entire device composed of the MN patch connected to the hand-made wireless potentiometric board.

The MN patch for pH sensing consists of two solid MNs acting as working (WE) and reference (RE) electrodes, that are fixed in a silicon rubber substrate. This configuration is particularly suitable to access to the wearable technology owing to its flexibility and biocompatibility. The manufacturing process is illustrated in Figure 2a. More details on substrate fabrication and MN fixation are provided in the Supporting Information. The WE is a potentiometric pH electrode based on a three-layer structure of carbon ink, functionalized multiwalled carbon nanotubes (f-MWCNTs) as an ion-to-electron transducer and a hydrogen-selective membrane (HSM) (see Figure 2b). The RE consists of a Ag/AgCl layer covered by a poly(vinyl butyral) reference membrane (RM) cocktail (see the Supporting Information), which provides a high and constant chloride concentration in the solid-state RE, as previously demonstrated elsewhere.^29,30^ The layer-by-layer structure of the RE is displayed in Figure 2c, whereas optical images of WE and RE are shown in Figure 2d,e, respectively. Transdermal pH measurements in euthanized rats were performed by coupling the developed MN patch with a portable potentiometer (Figure 2f). Note that all the rat-based experiments were carried out at the Karolinska University Hospital (Stockholm, Sweden) and assisted by the Operation Manager and Karolinska Experimental Research and Imaging Centre (KERIC) personnel. The rats were donated by KERIC and consisted of euthanized specimens that were previously used for other research purposes at KERIC. Importantly, the animals were not specifically euthanized for the purposes of our investigations but used as donated.

## Results and Discussion

The fabrication process for the pH WE was adapted from a recipe previously reported by our group.^26^ Thus, the deposition of the HSM was optimized considering two different approaches to incorporate the membrane cocktail: dip-coating and drop-casting. For both approaches, different membrane thicknesses were evaluated, which were obtained by changing the number of layers in dip-coating (3, 5, 10, 15, and 300) and both the number of layers (1–3) and volume per layer (0.5–3 μL) in drop-casting.

**Figure 3:**
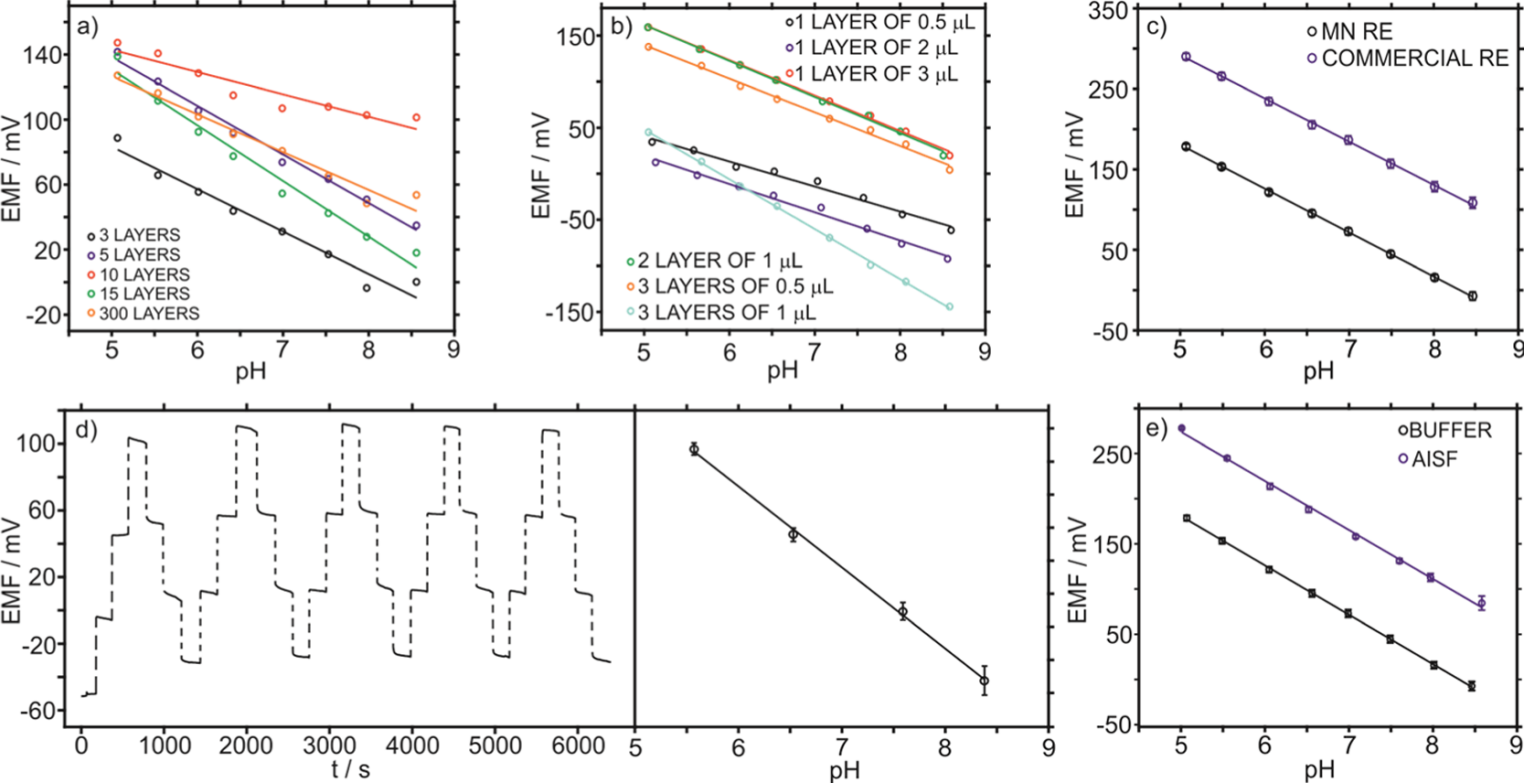
Calibration graphs obtained with two different deposition methods for the HSM incorporation: (a) dip-coating and (b) drop-casting. (c) Calibration curves obtained with the pH MN sensor against a commercial Ag/AgCl RE and the developed MN RE. (d) Dynamic response and average calibration graph for the carry-over test (ten consecutive calibrations by alternatively increasing and decreasing the pH in buffer solutions). (e) Calibration curves obtained with the MN patch in two different backgrounds: buffer solutions and AISF at increasing pH values.

Figure 3a,b depicts the calibration graphs obtained by separate buffer solutions and using electrodes prepared by each different deposition protocol. As observed in Figure 3a, none of the proposed configurations fabricated via dip-coating provided a Nernstian response, with the highest slope (34.3 mV) obtained using 15 layers of the membrane cocktail. Conversely, drop-casting deposition provided better results for both the slope and the linear range of response (LRR) (see Figure 3b). A Nernstian response was found for the configuration prepared by drop-casting three layers of 1 μL of the membrane cocktail. This MN sensor presented 54.4 mV dec^–1^ and 8.5–5.0 as slope and LRR, respectively. The differences between both methods are likely explained by a better control of the layers’ homogeneity by drop-casting rather than dip-coating, as suggested by images obtained using optical microscopy (see Figure S1 in the Supporting Information).

The optimized WE was combined with the MN RE to conform the final potentiometric MN-based patch. Notably, the performance of the MN RE was exhaustively characterized elsewhere, even proving its mechanical resilience during transdermal insertions.^26^Figure 3c compares the calibration graph (*n* = 3, three successive measurements using the same electrode) for the pH MN sensor against both a commercial Ag/AgCl RE (blue line) and the MN RE (black line). A very similar average slope was found in both cases: 54.6 ± 0.6 mV dec^–1^ for the MN RE and 53.8 ± 0.9 mV dec^–1^ for the commercial RE. An offset of 100 mV between both sensors was observed, which is evidently associated to the change of the reference electrode as reported elsewhere.^29^ As a result, the MN RE was used in all further measurements.

First, the analytical performance of the developed MN patch was characterized using an *in vitro* approach in buffer media. Between-electrode reproducibility was evaluated by carrying out one calibration using three different MN patches, obtaining a % RSD of 0.4 for the slope. Response repeatability, calculated from three consecutive calibration graphs performed with the same sensor, showed a % RSD of 2.5 and 4.1 for the slope and intercept, respectively. Reversibility was evaluated recording 10 successive calibration curves with increasing and decreasing pH values alternatively (Figure 3d). The sensor displayed a variation for the slope and the intercept of 3.5 and 2.4%, respectively, which reflect a rather good reversibility considering the entire experiment time scale (almost 2 h) and the rather large pH changes in the experiment. Fast response time (*t*_95_ < 5 s) and very low long-term drift (1.2 ± 2.1 mV h^–1^, in 10 mM HCl for a 16 h experiment, *n* = 3 sensors, see Figure S2a in the Supporting Information) were also achieved. Overall, in view of all these results, the pH MN patch presented excellent analytical features.

Next, the suitability of the MN patch for ISF analysis was evaluated through a selectivity study considering the major interferences found in this biological fluid, namely, Na^+^, K^+^, Mg^2+^, Ca^2+^, glucose, and urea. Table S1 collects the logarithmic selectivity coefficients (*n* = 3), calculated by the separate solution method.^31^ For all the interferences tested, selectivity coefficients were lower than the minimum value required for accurate measurements in ISF, which was estimated considering the highest concentration of each interference traditionally expected in ISF.^32^ Furthermore, the lack of synergistic effects between interferences was demonstrated by recording several calibration curves in AISF solution. Thus, Figure 3e presents the calibration graphs (*n* = 3) obtained in either buffer media (black line) or AISF (blue line). Similar slopes were observed in both media (54.2 ± 2.9 mV dec^–1^ in AISF and 54.6 ± 0.6 mV dec^–1^ in buffer) with the same LRR (from 8.5 to 5.0), which is indeed wide enough to cover expected pH values in ISF. Normal pH values in ISF range within 7.35 and 7.45 but may be altered due to the influence of diseases or health disorders/infections. For example, in the case of cancer, ISF is known to vary from 6.2 to 6.9.^33^ In addition, the long-term drift in AISF was rather acceptable (0.6 mV h^–1^ for 2 h experiment), as shown in Figure S2b in the Supporting Information.

Subsequently, *ex vivo* evaluation of the MN patch for transdermal pH detection in the ISF of different types of animal skins was accomplished to investigate two key aspects: (i) the response of the developed MN sensors is not altered and/or the external modification detached from the MN with the skin insertion; and (ii) appropriate accuracy of the measurements by means of a carefully designed validation protocol. One approach widely proposed in the literature to assess MN sensor resistance to skin penetration consists of using agarose hydrogels in contact with AISF to mimic the real skin system.^34,35^ The resiliency of MN sensors is evaluated by comparing the calibration parameters before and after the insertion into the hydrogel. Then, the suitability to use an external calibration for transdermal quantification of the analyte is assessed by measuring different concentrations in AISF that reach the hydrogel by diffusion. However, this approach does not provide a realistic evaluation of the ability of MN sensors to perform effective skin insertion because the hydrogel is softer and easier to penetrate than any real skin. In contrast, a closer approach to *in vivo* analysis utilizes animal skin instead.^26,36,37^ This methodology allows for a better evaluation of viscoelasticity and the adherence of undesired substances to the MN (including biofouling), thus providing close conditions to the further on-body use in animals and/or humans.

**Figure 4:**
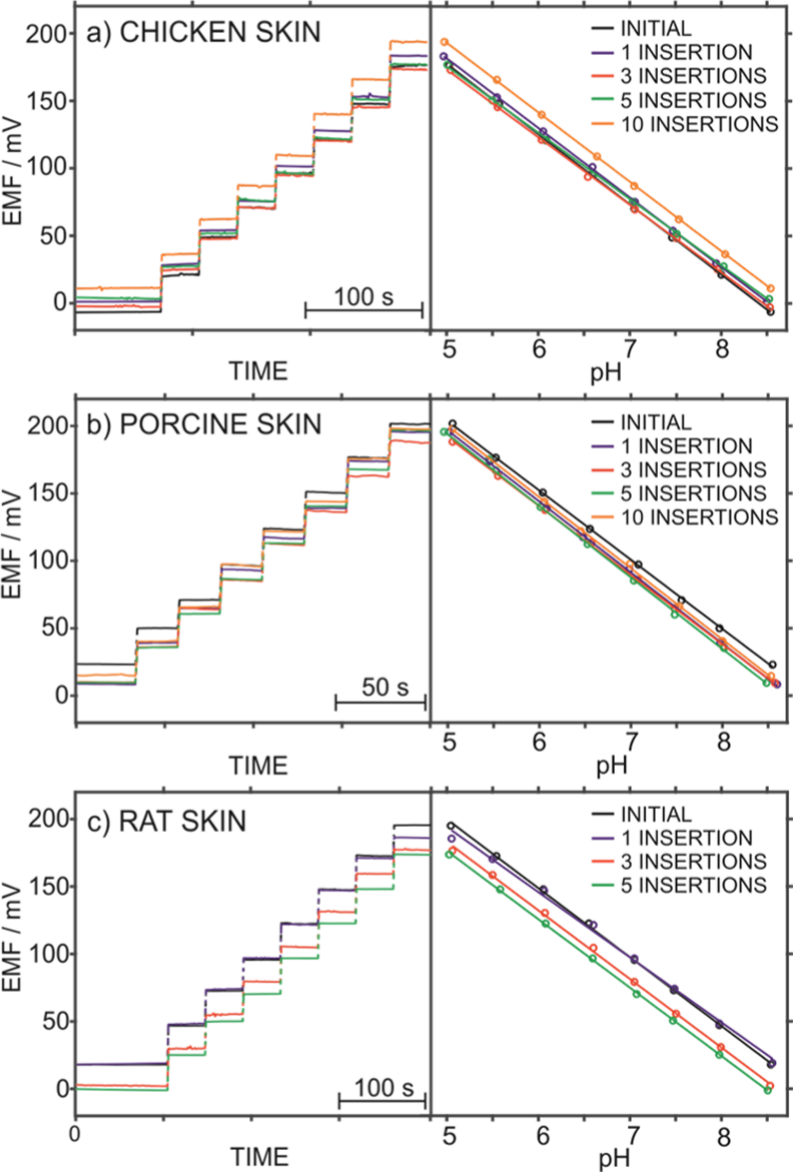
Dynamic responses and the corresponding calibration graphs observed for pH before and after several insertions into (a) chicken, (b) porcine, and (c) rat skin.

In this work, sensor resilience to skin penetration was assessed by manually inserting the MN patch in pieces of chicken, porcine, and rat skin several times (1, 3, 5, and 10 times) and registering the calibration curve in AISF afterward. This was compared with an initial calibration recorded prior to any insertion. Figure 4a shows the results obtained in chicken skin, which is the thinnest skin and the easiest to penetrate out of the three tested types of skin. No significant changes were observed in the slope after ten insertions (the initial slope was 52.0 mV dec^–1^ vs a slope of 51.4 mV dec^–1^ after ten insertions, a variation coefficient of 1.8%). Furthermore, if all the calibrations are considered, the obtained % RSD for the slope and intercept was 2.1 and 1.8, respectively. Thus, it is concluded that the response of the MN patch is not altered during insertion in chicken skin. Furthermore, the found RSD lies within the variation observed in the repeatability studies of the electrodes in the *in vitro* characterization (2.5 and 4.1 for the slope and intercept (*n* = 3) respectively). Accordingly, variations found in the standard electrode potential of the MNs with consecutive skin insertions can be likely attributed to an inherit variation of the sensor response rather than a perturbation due to the insertion cycles.

Analogous results were obtained in porcine skin (Figure 4b), with the initial slope of 51.3 mV dec^–1^ versus a slope of 52.7 mV dec^–1^ after ten insertions, % RSD of 1.5 and 1.2 for the slope and intercept considering all the calibration graphs. Significantly, the appropriate results found in porcine skin are particularly promising for future application of MN sensors in humans because this has striking similarities to the human skin in terms of general structure, thickness, hair follicle content, pigmentation, collagen, and lipid composition.^38^ Finally, Figure 4c displays the results obtained in rat skin. Similar calibration curves were also obtained after up to five insertions: with the initial slope of 51.1 mV dec^–1^ versus a slope of 50.7 mV dec^–1^ after five insertions and % RSD of 2.3 and 2.8 for the slope and intercept considering all the calibration graphs.

When using rat skin in the *ex vivo* studies (Figure 4c), the intercept was found to slightly shift to less positive values after each insertion. Although the variation after 5 insertions was acceptable, higher number of insertions would require a recalibration of the sensor to correct this drift. Notably, on-body experiments in rats herein presented are based in three skin insertions and the electrodes were calibrated before and after the transdermal measurements to minimize any error in the pH quantification arising from a change in the calibration graph.

The absence of any attachment of biological material in the MNs due to skin insertion and/or contact with the tissues was confirmed by microscopic images: a comparison of the images taken before (Figure S1b) and after (Figure S1c) penetration in chicken skin is presented in the Supporting Information. As observed, no particles or pieces are attached to the MN sensor after the insertion. In addition, no sign about the deterioration and/or detachment of the sensing element was detected. This confirms, in turn, the absence of marked variations in the electrode calibration after several insertions (Figure 4a), hence pointing out the absence of biofouling effects.

The second part of the *ex vivo* assays focused on the validation of transdermal pH measurements with the developed MN patch once inserted in animal skin and therefore demonstrating the suitability of an external calibration of the sensor for the pH quantification in further on-body studies. We designed these tests on the basis of skin pieces that were conditioned 24 h at different pH. This experiment is not trivial, and one should not assume that the intradermal concentration of any analyte can be easily modified just by direct contact of the skin with an external solution of the desired analyte concentration. For example, Senel et al. reported a lower chronoamperometric response for urea when MN sensors were measuring inside phantom gel than that expected according to the concentration fixed in external AISF.^39^ This behavior was attributed to the slow diffusion of urea in phantom gel. Similar conclusions were drawn in our previous studies regarding the determination of K^+^ in chicken skin using potentiometric MN sensors.^26^ It was demonstrated that the lower potassium concentration determined inside the skin as compared to the concentration in the AISF solution that soaked the skin was due to the diffusion and distribution of the ion driven by the concentration gradient at the skin–AISF interface.^26^

**Figure 5:**
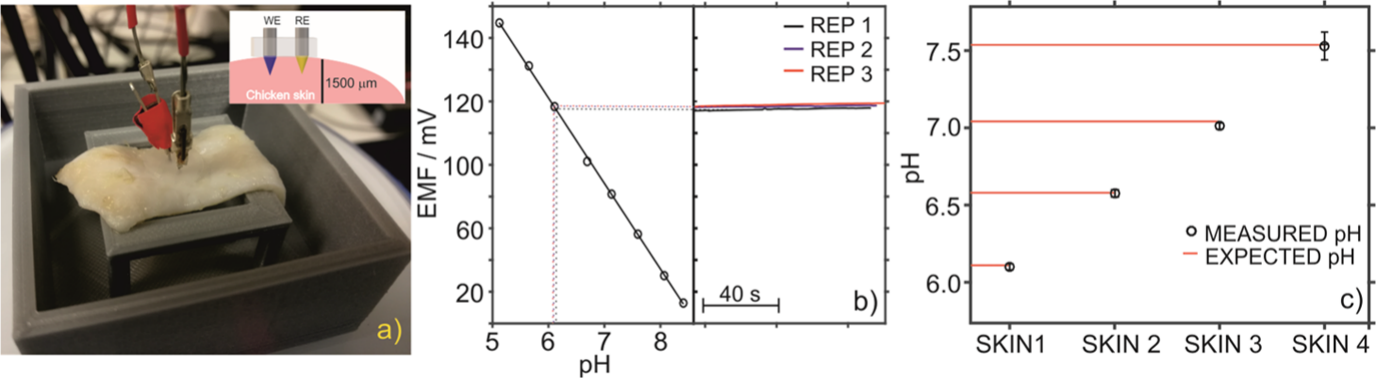
(a) Setup for *ex vivo* measurements performed with the MN patch inserted in chicken skin conditioned at different pH values. Inset: scheme of the MN insertion. (b) External calibration graph and three consecutive potentiometric responses obtained during the measurement of chicken skin at pH 6.0. (c) Measured and expected values in *ex vivo* pH measurements of four chicken skin pieces conditioned at different pH values.

In this paper, we opted for performing experiments by conditioning the pieces of animal skin with a known analyte concentration for a 24 h process and then measuring the transdermal pH with the MN patch (Figure 5a). Alternatively, the concentration inside the skin can be measured by mincing and digestion followed by analysis with reference analytical techniques.^37^ However, this option is not suitable for the particular case of pH determination because acid digestion will affect the measurement. To confirm that 24 h conditioning is enough to reach the desired pH inside the skin, we collected the ISF by means of a custom-made system based on hollow MNs and measured the pH with a commercial micro-pH electrode. The collection device was based on a commercially available hub with hollow MNs connected to a peristaltic pump (see the Supporting Information for more details). We found that the pH in the ISF was very similar to that fixed in the conditioning solution (see Table S2 in the Supporting Information). In addition, the pH of the conditioning solution was measured by means of a commercial pH meter to ensure that pH was retained during 24 h.

Transdermal potentiometric responses inside each type of skin were recorded by triplicate and pH values were calculated by extrapolating average potentials into an external calibration performed in AISF (see Figure 5b). Figure 5c displays the results obtained for four chicken skin pieces conditioned at different pH values, with black circles representing the values measured by the MN patch and red lines indicating the expected pH values. The accuracy and precision of the transdermal measurements using the MN results in the *ex vivo* experiments are the perfect preamble for further animal-based tests based on the on-body use of the new MN patch.

Prior to any *in vivo* tests in rats and humans, it is mandatory to demonstrate the biocompatibility level of the MN patch ensuring that no toxic effect is caused by direct contact, leaching, or detachment of the sensing element. In this context, we have recently reported on the cytotoxicity of all the materials used in the pH MN sensor herein developed.^26,28^ Briefly, cell viability and cell proliferation studies using fibroblasts were carried out with bare MNs, MNs coated with either carbon or Ag/AgCl ink, f-MWCNT, and the HSM. In all cases, after 96 h of incubation, the total number of counted cells was comparable to control conditions (fibroblasts immersed in culture media and without the presence of material/compound), indicating the absence of cytotoxicity effects. However, this outcome should not be generalized to any MN-based ISE because, unlike HSM, other membrane compounds, such valinomycin-based membranes for potassium or nonactin membranes for ammonium, revealed a certain level of cytotoxicity after 96 h.^28^

Then, *in vivo* tests should come accompanied by validated measurements using a gold standard technique, although the literature is really scarce in this issue. Indeed, the implementation of this validation is not trivial due to the difficulty of extracting enough volume of the same ISF that is measured by the MN. We explored two different validation protocols for the pH MN tests in rats. The first approach measures subcutaneous pH by means of a commercial pH microelectrode after opening the specimen. The second protocol is based on ISF extraction using a home-made system based on a commercially available hollow MN hub (see the Supporting Information for more details). Interstitial pH was measured in seven rats with different genders and ages (see Table S3 in the Supporting Information) using the newly developed MN patch and the two validation protocols. All measurements were performed sequentially in one rat before moving to the next one to minimize pH changes due to evaporation and/or stop in blood flow. Notably, the rats were donated by KERIC and consisted of euthanized specimens that were previously used for other research purposes at KERIC.

**Figure 6:**
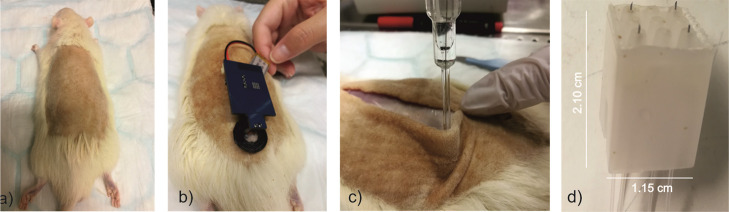
Pictures of on-body measurements in a euthanized rat specimen: (a) shaved rat prepared for the insertion of the MN patch. (b) pH MN patch coupled with the potentiometric electronic board and providing transdermal measurements in the rat back. (c) Subcutaneous measurement of pH using a micro-pH meter. (d) Home-made tool for ISF collection based on a hollow MN hub connected to a peristaltic pump.

To implant the MN sensors, the back of the rats was shaved to facilitate MN insertion and visual inspection (Figure 6a). Subsequently, the MN patch was inserted in the rat back and secured with a ring of polyurethane (PU) to prevent any movement of the MNs during the on-body measurements. Finally, the patch was connected to a wireless potentiometric board and the response was recorded until the steady-state potential was reached, 40 s approx. (see Figure 6b). Three consecutive insertions were performed to obtain the measurement in triplicate and to confirm that the sensing element is not detached from the MN.

A total of 7 rats were tested, with the rat number 5 monitored with two different (and twin) MN patches (Table S3). In none of the cases, we could visually detect any modification of the MN after transdermal measurements. No significant alteration was detected in the potentiometric response upon increasing insertions either. Moreover, we found variation coefficients of less than 1% in all the tested rats, except for the first measurement round in rat number 5 and in rat number 6, where ca. 5% of variation was observed. This higher variation in triplicate measurements specifically obtained in two specimens is likely associated to the quality of the hand-made fabricated MN patches: the three measurements accomplished in rat 5 provided values (in this order) of 7.51, 7.03, and 6.60. Then, when measurements were repeated in the same rat but with a different MN patch, the provided values were 6.36, 6.48, and 6.48, with a variation coefficient in the range of the 1% as in the majority of the rats. Thus, in the case of the first measurement round, the real pH is seemingly within the two last values, with the very first measurement interpreted as an outlier.

After the transdermal measurements, the first validation method was carried out by means of an incision in the rat’s back, exposing the subcutaneous ISF and measuring the pH with a commercial microelectrode (Figure 6c). For the second validation method, ISF was directly extracted from the rat back with the MN hub (Figure 6d, see the Supporting Information for more details). ISF extraction was prolonged for 30 min, obtaining significantly different sample volumes for each rat, ranging from nonmeasurable volume (almost zero) to more than 100 μL (Table S3 in the Supporting Information). The pH of the collected ISF was measured with an ultramicro pH electrode, specifically designed for low-volume sample analysis (∼0.5 μL). Only for one rat, it was not possible to extract enough ISF to be measured with the pH meter. Interestingly, the highest volumes of ISF were extracted from the youngest rats (2 months), which is likely attributed to both smoother skin and higher hydration level. This combination enhances hydraulic conductivity of tissue, leading to spacing tissue fibers that facilitate ISF extraction.^40^

**Figure 7:**
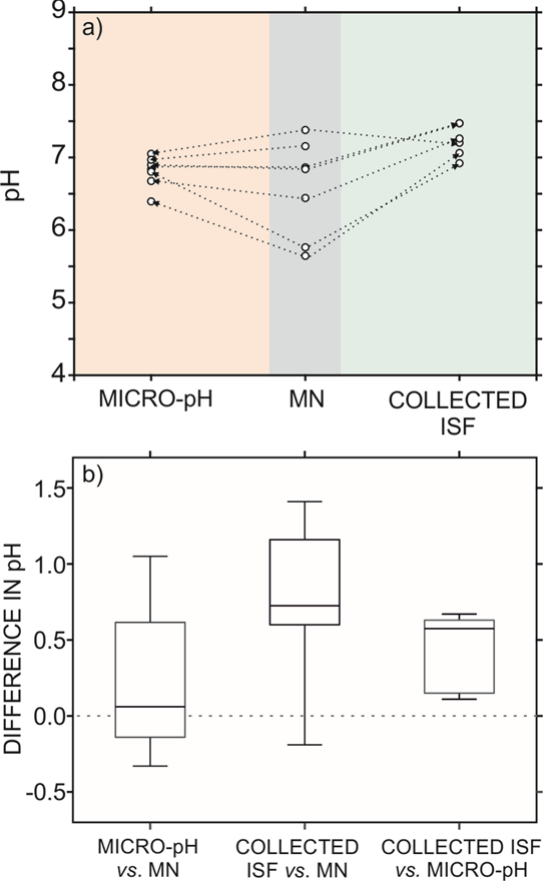
(a) Comparison of pH measurements with the MN patch and two different validation procedures: subcutaneous pH measurements with the micro-pH meter and ISF extraction. Connected lines represent values obtained for the same rat. (b) Paired sample *t*-test box plot representing statistically analyzed differences in pH measurements with the MN patch, subcutaneous data, and ISF extraction. Gray dotted line represents the null hypothesis.

Individual correlations between the three types of measurements were statistically evaluated to quantify the accuracy of the pH MN patch: separate paired sample *t*-tests (also known as the dependent sample *t*-test) were carried out. Notably, since measurements performed on the same rat are treated as a single pair of observation, differences in pH among different rats will not influence the test. Graphical representations of the paired sample *t*-test are displayed in Figure 7, with Figure 7a presenting the direction in the variation of the pH measurements in each single rat (comparing the MN with each validation strategy), and Figure 7b being a box and whisker plot of the measured differences between the three methods. Attending first to the comparison of MN results with subcutaneous measurements and considering a 95% of confidence level, the *t*_calc_ = 1.2 was lower than *t*_stat_ = 2.5 and hence there were no statistically meaningful differences between both methods (accepting the null hypothesis, see Figure 7b). This result is easily visualized in Figure 7a, where no clear direction in variation appears. Contrarily, the paired sample *t*-test indicated that there was a statistically significant difference between MN results and the pH measurements in the collected ISF (*t*_calc_ = 3.27 > *t*_stat_ = 2.57).

A further comparison between the pH provided by MNs and the subcutaneous measurements can be accomplished by directly comparing the differences in values (see Table S3). In all the cases, this difference is lower than 0.3 pH units, except for rat number 4 and number 7 (ca. 1 pH unit). Despite the results being within the bounds of experimental variance, ISF evaporation is hard to be controlled in subcutaneous measurements when the skin is exposed to the air, and this may result in significant variations compared to the MN data. Transdermal measurements are indeed advantageous compared with the two validation techniques, which is required for sample and/or specimen manipulation. On the other hand, as observed in Figure 7a, ISF extraction tends to provide pH values higher than the MN patch. Thus, the two validation methods are not providing the same information and evidently, at least one of them presents some systematic error. Moreover, comparison of the two validation methods results in *t*_calc_ = 4.4 > *t*_stat_ = 2.6, meaning that the results are statistically different (see Figure 7b). A plausible explanation of this result is that ISF extraction takes place for a long time period (30 min) in which blood flow has been stopped because the rat is no longer alive, therefore affecting normal diffusion processes between blood and ISF.

The same observation was reported by Mani et al. for anaesthetized mice: pH values measured with MN sensors in CSF and bladder were slightly lower than those measured using commercial pH electrodes after fluid extraction.^16^ Authors attributed the observed differences to a change in the sample pH caused by the removal of biological fluids from their buffered (physiological) conditions. Also, subcutaneous measurements are directly accomplished in the rat, and, therefore, less marked alterations in the sample are expected. As a result, subcutaneous measurements seem to be more reliable as the validation procedure. Nevertheless, it should be noted that this method requires surgery, and therefore, future efforts should be devoted to the development of noninvasive and reliable validation protocols toward an adequate validation of pure *in vivo* measurements in animals and humans.

## Conclusions

A fully validated potentiometric pH MN sensor for transdermal pH measurements in ISF has been presented. This is the first time that such a sensor is demonstrated for on-body measurements in rats after a deep *in vitro* and *ex vivo* characterization at the laboratory scale. Stainless-steel solid MNs were modified following a layer-by-layer approach to provide robust and reliable pH measurements. Analytical characterization of the MN patch under *in vitro* conditions demonstrated suitable Nernstian response, excellent repeatability and reproducibility, fast response time, adequate drift, and an LRR wide enough to cover both physiological and abnormal pH levels in ISF. *Ex vivo* assays using chicken, porcine, and rat skin revealed that the new MN patch is resilient to skin insertion and provides high precision and accuracy, as inferred from an *ex vivo* validation using preconditioned pieces of animal skin. The MN patch was successfully implemented into on-body assays in rats, measuring interstitial pH in seven specimens. Two different validation protocols were evaluated, concluding that subcutaneous pH measurements are more reliable than the analysis of extracted ISF samples. Overall, this work provides a clear guide on how to properly characterize and validate the analytical performance of newly developed MN sensors for any type of analyte (ions and biomolecules), starting from *in vitro* conditions and reaching *on-body* measurements. This is indeed a key aspect in making meaningful advances in the development and application of wearable eHealth devices based on minimally invasive MNs.

## Supporting Information Available

Experimental details, logarithmic selectivity coefficients for the pH MN electrode; pH monitoring of chicken skin conditioned for 24 h; results obtained in rat assays via MNs, micro pH meter, ISF collection; and optical microscopic images of pH MN sensors (PDF)
