# Supplementary material for: Toward In Vivo Transdermal pH Sensing with a Validated Microneedle Membrane Electrode
Source: ACS Sens. 2021 Feb 10;6(3):1129–37. doi: 10.1021/acssensors.0c02397 (PMC8023800; doi:10.1021/acssensors.0c02397)
Supplement: Supplementary file 1 — se0c02397_si_001.pdf [file se0c02397_si_001.pdf]

Supporting information for:

Towards In Vivo Transdermal pH Sensing with Validated Microneedle Membrane Electrode

Juan José García-Guzmán, Clara Pérez-Ràfols, María Cuartero, Gaston A. Crespo*

Department of Chemistry, School of Engineering Science in Chemistry, Biochemistry and Health, Royal Institute of Technology, KTH. Teknikringen 30, SE-100 44, Stockholm, Sweden.

(*) Corresponding author: gacp@kth.se

Experimental Section

Reagents, materials and instrumentation. Multiwalled carbon nanotubes (MWCNTs), hydrogen ionophore I (selectophore grade), sodium tetrakis[3,5-bis(trifluoromethyl)phenyl]borate (NaTFPB, > 98 % purity), bis(2-ethylhexyl)sebacate (DOS, ≥ 97 % purity), polyurethane (PU), tetrahydrofuran (THF, ≥ 99 % purity) and polyvinyl butyral (PVB) were purchased from Sigma-Aldrich. Analytical grade chloride salts of calcium, magnesium and sodium as well as sodium carbonate, sodium phosphate, sodium citrate, bovine serum albumin (BSA), urea and glucose were purchased also from Sigma-Aldrich. Silver/silver chloride (Ag/AgCl) paste 50/50 (C2131007D3) and carbon ink (C2030519P4) were obtained from Sunchemical (UK).

A mixture of 1 wt.% hydrogen ionophore I (19 mmol/kg), 0.76 wt.% NaTFPB (8.6 mmol /kg), 33 wt.% PU and 65 wt.% DOS in 1 mL of THF was used to prepare the cocktail for the hydrogen-selective membrane (HSM).[1] The reference membrane cocktail was prepared by dissolving 78 mg of PVB and 50 mg NaCl in 1 mL of methanol as previously described in literature.[2]

All solutions were prepared in 18.2 MΩ cm^{-1} doubly deionized water (Milli-Q water systems, Merck, Millipore). Artificial interstitial fluid was prepared according to the recipe previously described in literature.[3, 4] Briefly, the artificial interstitial fluid composition was 20 g L^{-1} BSA, 3.5 mM KCl, 1.5 mM $CaCl_2$, 0.7 mM $MgCl_2$, 140 mM NaCl, 26 mM $NaHCO_3$, 1.7 mM Na_2HPO_4, 6 mM glucose and 7 mM urea.

Silicon rubber (Ecoflex 00-50 platinum cure, USA) and solid stainless-steel microneedles (MN) (Dermaroller local supplier, Sweden) were employed for the fabrication of the MN patch. The microscopic images were obtained by a homemade Cerna microscope (Thorlabs, USA).

All the experiments involving rats were performed at the Karolinska University Hospital (Sweden) and assisted by the operation manager and Karolinska Experimental Research and Imaging Centre (KERIC) personnel, using specimens that were euthanized for other researcher purposes rather than those in the presented paper. Porcine and chicken skin were purchased from a local supermarket (Stockholm, Sweden). Porcine, skin and rat skin were cut into circled 6 cm^2 area and stored in the freezer at -18°C. Before being used in ex vivo experiments, each piece of skin was thoroughly cleaned with distilled water. For the ex vivo measurements of pH in conditioned skin, skin pieces were immersed in a buffer solution with the selected pH and stored in the fridge at 2°C for 24 hours prior to the assay.

Electromotive force (EMF) was measured with a high input impedance (10^{15}) EMF 16 multichannel data acquisition device (Lawson laboratories, INC) against a double junction Ag/AgCl/sat. KCl/1M LiOAc reference electrode (6.0726.100, Metrohm Nordic Sweden) or the MN-based reference electrode (see below). A hand-made potentiometric board with wireless data transmission was used for the rat-based experiments in Karolinska Institute.

Fabrication of the MN patch. The MN patch consists of a silicon rubber substrate with inserted MN-based WE and RE. The substrate is fabricated by mixing equal volumes of solutions labelled as 'Part A' and 'Part B' of the commercial pourable silicon rubber and filling a 3D printed mould of 13 mm of diameter and 1 mm of depth with the resulting mixture. Afterwards, the substrate was allowed to cure for 3 hours, according to the manufacturer instructions. The MNs used in these experiments have a full length of 1.5 mm and a diameter of 400 µM. The modification process to obtain the WE and RE is schematized in **Figure 2** in the main manuscript. Initially, the stainless-steel solid MNs were coated with the

corresponding ink (carbon for the working electrode and Ag/AgCl for the reference electrode). Secondly, the MNs were inserted in the substrate and glued with Loctite Super Glue (Henkel Norden AB), which was allowed to dry at room temperature for 4 h. Finally, the functionalization with the suitable membranes was performed for each MN.

Preparation of the WE. First, the stainless-steel solid MN was coated for 5 s with the carbon ink and the created film was allowed to cure in the oven for 10 min at 120 ºC. After proper fixation in the substrate, the f-MWCNTs film was then formed by drop casting (10 layers of 2 µL) a 1 mg mL^{-1} solution of f-MWCNTs in ethanol onto the modified MN, with 4 min drying at room temperature in-between layers. Finally, 3 layers of 1 µL of the pH membrane cocktail were drop casted on top of the f MWCNTs film. In this case, each layer was allowed to dry for 20 min before the addition of the following one, and the final layer was dried at room temperature for 4 h before conditioning the electrode overnight in 1 mM HCl solution.

Preparation of the RE. A pseudo-RE was first obtained by dip coating a stainless-steel solid in Ag/AgCl commercial ink[5]. This layer was then cured in the oven (120 ºC, 10 min). After appropriate fixation in the substrate, poly(vinyl butyral) (PVB) reference membrane cocktail was drop casted (3 µL x 3 times) on top of the Ag/AgCl film. Each layer was allowed to dry for 20 min before drop casting the next one. Next, the last layer was dried for 4 h at room temperature before the overnight conditioning in 3 M KCl. Finally, the RE was dried at room temperature for 1 h and 2 µL of polyurethane were drop casted on top of the MN and left to dry in air for 4 h. This last step enhances the potential stability of the RE and hinders the salt leaching out of the RE.[6]

***Potentiometric measurements*.** Calibration experiments were carried out at room temperature (22±1 °C) and under constant stirring of 500 rpm (stirrer IKA COLOR SQUID S000, IKA, Germany). The MN-based electrodes (WE and RE) were connected to the potentiometer by a cable based on electrical clamps and BNC outputs. In the case of rat-based experiments, a hand-made potentiometric board was used. The MNs were connected by copper tape.

Selectivity studies. The activity coefficients were calculated using a two-parameters Debye-Hückel approximation from the experimental concentrations.[7] Each logarithmic activity was then plotted against the corresponding steady-state potential and the data were fitted to the Nernst equation.[8] Selectivity was evaluated using the separate solution method according to Bakker et al.:[9] Individual calibration graphs were accomplished for the primary and the interfering cations (Mg^{2+}, Ca^{2+}, Na^{+} and K^{+}) and the logarithmic selectivity were calculated by extrapolating the response to a_I = 1 M using the portion of the calibration curve close to Nernstian response.

Ex vivo assays. In order to test the mechanical resilience of the MN patch, a test consisting of several calibration graphs before and after a certain number of insertions in different type of animal skin (1, 3, 5 and 10) was carried out (**Figure 4** in the main manuscript). Accuracy was evaluated by measuring portion of chicken skin previously conditioned at different pHs. The conditioned skin was fixed in a polylactic acid (PLA) 3D printed holder (**Figure 5** in the main manuscript) and then, the MN patch was inserted in the skin to register the potential and obtain the transdermal pH via comparison with a previous calibration accomplished in the beaker according to the general calibration procedure described above.

Transdermal detection of pH in interstitial fluid in rats. Bio-breeding diabetes-prone rats (BBDP-rats) euthanized (CO_2 chamber) for other research purposes and donated by KERIC were employed in this study. In particular, the specimens weighed 270-280 g each. The rats' backs were shaved (**Figure 6a** in the main manuscript) and previously calibrated MN patches

were inserted through the skin. The potential was recorded with a hand-made potentiometric electronic board equipped with a bluetooth low energy system able to take up to 900 samples per second (see **Figure 6b** in the main manuscript). After this, the rats' backs were opened with a scalpel, exposing the subcutaneous tissue. ISF in this tissue was measured *in situ* with a micro-pH meter (LL, biotrode, Metrohm, Nordic Sweden) for validation purposes (**Figure 6c** in the main manuscript). Additionally, ISF was extracted from the skin by using a homemade device consisting in a plastic hub containing 4 hollow microneedles (0.24 x 0.11 x 10 mm, Micropoint Technologies Pte Ltd, Singapore). The hollow length exposed in the hub was 1.5 mm and it was coupled with a microfluidic PFTE tubing (Sigma-Aldrich) with 0.3 and 0.6 mm of inner and outer diameter, respectively (**Figure 6d** in the main manuscript). A time of 30 min extraction was assisted by means of a tubing (Tygon LMT-55, ISMATEC, Cole-Parmer GmbH, Germany) connected to a peristaltic pump (ISMATEC IPC series, Cole-Parmer GmbH, Germany). ISF extraction was performed just after MN sensing to minimize the possible alterations of the ISF either in quantity or quality given the fact that the rat is no longer alive. In most of the cases, 30 min were necessary to extract a useful amount of ISF for further studies. Lastly, the pH of the collected ISF samples was measured with an ultra-micro pH meter (Orion, Ultra-Micro Combination pH Electrode, Thermo Scientific).

Tables

Table S1. Logarithmic selectivity coefficients for the pH MN electrode.

	MN electrode	Expected Concentration (mM)[3, 4, 10, 11]	Minimum required value[a]
$\log K_{H,Na}^{pot}$	-6.0 ± 0.09[b]	134.6-146.5	-3.5
$\log K_{H,K}^{pot}$	-6.7 ± 0.08[b]	3.17-3.50	-1.8
$\log K_{H,Ca}^{pot}$	-5.0 ± 0.09[b]	1.18-1.54	-2.9
$\log K_{H,Mg}^{pot}$	-10.7 ± 0.17	0.66-0.72	-3.2
$\log K_{H,urea}^{pot}$	-3.5 ± 0.03[b]	5.4-6.2	-2.2
$\log K_{H,glucos}^{pot}$	-3.42 ± 0.01[b]	4.4-6.0	-2.3

[a]Values calculated from the higher concentration of the interferent expected in interstitial fluid. [b]These cations did not display a Nerstian slope and therefore, the calculated logarithmic selectivity coefficients are "biased" and can be only interpreted qualitatively as 'apparent' values.[8]

Table S2. pH monitoring of chicken skin conditioned for 24 h.

Initial buffer pH	pH in soaking solution[a]	pH inside the skin[b]
7.53	7.52	7.49
7.00	6.97	6.99
6.55	6.60	6.61
6.10	6.17	6.23

[a]pH value obtained via direct measurement with micro pH meter in the solution soaking the skin after 24-h; [b]pH value obtained with micro pH meter in the solution extracted from the chicken skin by the homemade tool.

Table S3. Characteristics of the rats employed in *in vivo* assays and the corresponding pH values obtained via MNs, micro pH meter and ISF collection.

Rat	Age (months)	Sex[a]	pH MN	Subcutaneous pH[b]	MN–Subcutaneous (Abs difference)	Collected ISF pH[c]	Collected Volume (µL)
1	12	F	6.86 ± 0.01	6.84	0.02	7.47	7
2	5	M	6.84 ± 0.06	6.90	0.06	7.47	12
3	5	M	7.38 ± 0.06	7.05	0.33	7.20	5.5
4	2	M	5.65 ± 0.10	6.39	0.74	7.06	>100
5	2	M	7.05 ± 0.37	6.68	0.37	7.26	>100
5	2	M	6.44 ± 0.05	6.68	0.24	7.26	>100
6	5	F	7.15 ± 0.22	6.97	0.18	–	none
7	5	F	5.76 ± 0.03	6.81	1.35	6.92	5.5

[a]Rat sex: Female (F) or male (M); [b]pH value obtained via direct measurement with micro pH electrode in a surgically open rat; [c]pH value obtained with ultra-micro pH electrode after ISF extraction with hollow MNs.

Figures

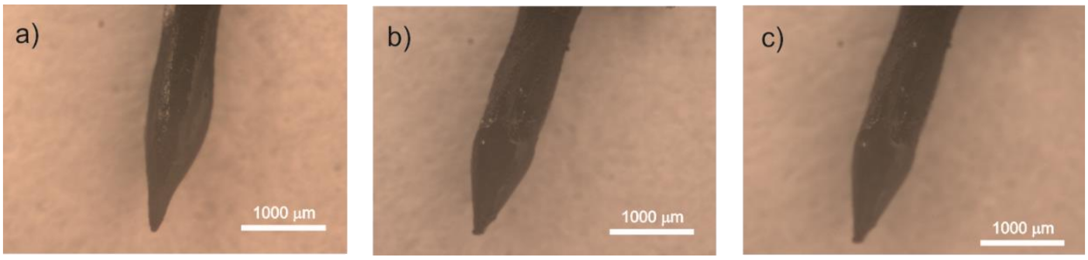

Figure S1. Optical microscopy images of pH MN sensors prepared using different membrane deposition protocols: **(a)** 3 layers of dip coating, with 20 min drying in-between layers and 4 h drying at room temperature after the last layer; and **(b)** drop casting 3 layers of 1 µL each, with 20 min drying in-between layers and 4 h drying at room temperature after the last layer. **(c)** Optical microscopy images of pH MN sensors prepared as in (b) after 1 insertion in chicken skin.

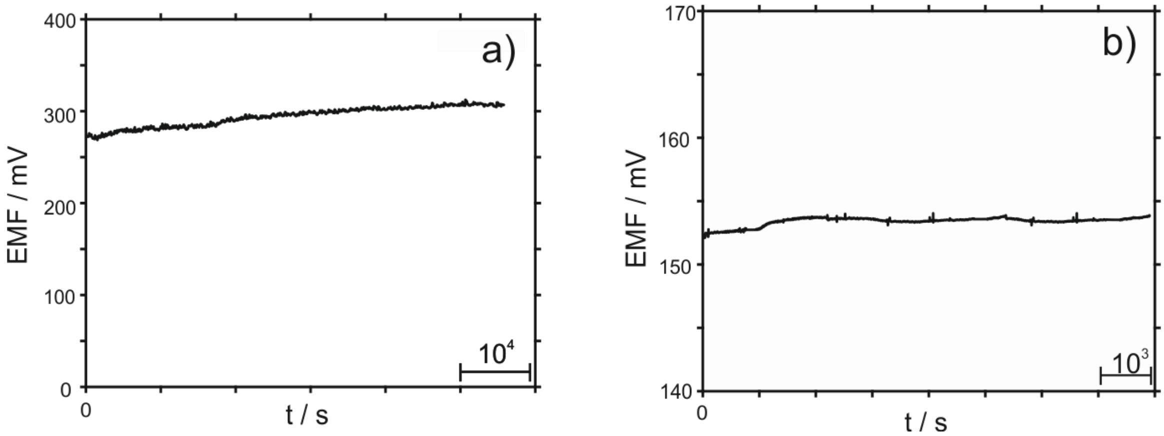

Figure S2. (a): Tame trace of the potentiometric response of pH MNs: (a) in HCl solution 10 mM during 16 h, (b) in AISF during 2h.

References

1. Canovas, R.; Padrell Sanchez, S.; Parrilla, M.; Cuartero, M.; Crespo, G. A., Cytotoxicity study of ionophore-based membranes: toward on-body and in vivo ion sensing. *ACS Sens.* **2019,** *4* (9), 2524-2535.
2. Guinovart, T.; Crespo, G. A.; Rius, F. X.; Andrade, F. J., A reference electrode based on polyvinyl butyral (PVB) polymer for decentralized chemical measurements. *Anal Chim Acta* **2014,** *821*, 72-80.
3. Bretag, A. H., Synthetic interstitial fluid for isolated mammalian tissue. *Life Sciences* **1969,** *8* (1), 319-329.
4. Fogh-Andersen. Niels; M.Altura, N.; T.Altura, B.; Siggard-Anderssen, O., Composition of Interstitial Fluid. *General Clinical Chemistry* **1995,** *41* (10), 1522-1525.
5. *Handbook of Reference Electrodes*. Springer-Verlag Berlin Heidelberg: Berlin, 2013; Vol. 1, p 344.
6. Parrilla, M.; Canovas, R.; Jeerapan, I.; Andrade, F. J.; Wang, J., A Textile-Based Stretchable Multi-Ion Potentiometric Sensor. *Adv Healthc Mater* **2016,** *5* (9), 996-1001.
7. Meier, P. C., Two parameter Debye Hückel approximation for the evaluation of mean activity coefficients of 109 electrolytes. *Analytica Chimica Acta* **1982,** *136*, 363-368.
8. Bakker, E.; Pretsch, E., Modern potentiometry. *Angew Chem Int Ed Engl* **2007,** *46* (30), 5660-8.
9. Bakker, E. P., E.; Buhlmann, P., Selectivity of Potentiometric Ion Sensors. *Analytical Chemistry* **2000,** *72*, 1127-1133.
10. Krogstad, A. L.; Jannsson, P. A.; Gisslén, P.; Lönnroth, P., Microdialysis methodology for the measurement of dermal interstitial fluid in humans. *Br. J. Dermatol.* **1996,** *134*, 1005-1012.
11. Strindberg, L.; Lönnroth, P., Validation of an endogenous reference technique for the calibration of microdialysis catheters. *Scand. J. Clin. Lab. Investig.* **2009,** *60* (3), 205-212.
